# Renal Cell Carcinoma Perfusion before and after Radiofrequency Ablation Measured with Dynamic Contrast Enhanced MRI: A Pilot Study

**DOI:** 10.3390/diagnostics8010003

**Published:** 2018-01-08

**Authors:** Tze Min Wah, Steven Sourbron, Daniel Jonathan Wilson, Derek Magee, Walter Martin Gregory, Peter John Selby, David L. Buckley

**Affiliations:** 1 Department of Diagnostic and Interventional Radiology, Institute of Oncology, Leeds Teaching Hospitals Trust, St. James’s University Hospital, Beckett Street, Leeds LS9 7TF, UK; 2 Division of Medical Physics, University of Leeds, Leeds LS2 9JT, UK; S.Sourbron@leeds.ac.uk (S.S.); D.L.Buckley@leeds.ac.uk (D.L.B.); 3 Department of Medical Physics, St. James’s University Teaching Hospital, Beckett Street, Leeds LS9 7TF, UK; Daniel.Wilson8@nhs.net; 4 School of Computing, University of Leeds, Leeds LS2 9JT, UK; D.R.Magee@leeds.ac.uk; 5 Professor of Statistical Methodology in Clinical Trials, Clinical Trials Research Unit (CTRU), University of Leeds, Clinical Trials Research House 71-75 Clarendon Road, Leeds LS2 9PH, UK; W.M.Gregory@leeds.ac.uk; 6 Professor of Cancer Medicine, Leeds Institute of Molecular Medicine, St. James’s University Hospital, Beckett Street, Leeds LS9 7TF, UK; P.J.Selby@leeds.ac.uk

**Keywords:** radiofrequency ablation, quantitative measurement, perfusion, MRI, renal cell carcinoma

## Abstract

Aim: To investigate if the early treatment effects of radiofrequency ablation (RFA) on renal cell carcinoma (RCC) can be detected with dynamic contrast enhanced (DCE)-MRI and to correlate RCC perfusion with RFA treatment time. Materials and methods: 20 patients undergoing RFA of their 21 RCCs were evaluated with DCE-MRI before and at one month after RFA treatment. Perfusion was estimated using the maximum slope technique at two independent sittings. Total RCC blood flow was correlated with total RFA treatment time, tumour location, size and histology. Results: DCE-MRI examinations were successfully evaluated for 21 RCCs (size from 1.3 to 4 cm). Perfusion of the RCCs decreased significantly (*p* < 0.0001) from a mean of 203 (±80) mL/min/100 mL before RFA to 8.1 (±3.1) mL/min/100 mL after RFA with low intra-observer variability (*r* ≥ 0.99, *p* < 0.0001). There was an excellent correlation (*r* = 0.95) between time to complete ablation and pre-treatment total RCC blood flow. Tumours with an exophytic location exhibit the lowest mean RFA treatment time. Conclusion: DCE-MRI can detect early treatment effects by measuring RCC perfusion before and after RFA. Perfusion significantly decreases in the zone of ablation, suggesting that it may be useful for the assessment of treatment efficacy. Pre-RFA RCC blood flow may be used to predict RFA treatment time.

## 1. Introduction

Increased renal cell carcinoma (RCC) detection is due to the wider use of radiological imaging and also related to increased incidence of RCC in the general population [1]. The majority of incidentally detected RCCs nowadays are at T1 stage [2]. Therefore, increasingly, the general consensus for the treatment of smaller RCCs (<4 cm) is to offer minimally invasive therapies e.g., nephron sparing surgery or image guided radiofrequency ablation (RFA) or cryoablation [3].

Today, imaging is vital in the pre-procedural planning, intra-procedural monitoring and post-procedural assessment of treatment efficacy of RFA [4,5]. The treatment efficacy is usually assessed with triple phase contrast enhanced Computerised Tomograpgy (CT) or Magnetic Resonance Imaging (MRI) at 1, 3, 6 and 12-monthly and annually thereafter for a 5–10 year period, depending on the local cancer network policy [4,5]. In our institution, we routinely use dynamic contrast enhanced (DCE) MRI to monitor treatment efficacy in the zone of ablation. The advantages of MRI are that it has good spatial and contrast resolutions without an ionizing radiation burden. Typically, the lack of contrast enhancement in the zone of ablation is usually interpreted as successful ablation [5]. However, the zone of ablation can still exhibit some enhancement and it is this variability that can pose a clinical dilemma when deciding whether there is complete tumour cell death [6,7]. Furthermore one recent study has shown that qualitative imaging is not infallible with detection of viable disease of the non-involuting zone of ablation even when imaging shows no significant enhancement post-RFA [8].

In clinical practice, the ability to perform quantitative assessment of RCC perfusion may potentially facilitate the treatment planning of renal tumours by predicting the treatment time and monitoring the treatment efficacy post-RFA. For the planning of RFA treatment, this ensures an adequate scheduled time allocation. The hypothesis being that a better perfused tumour will take longer to treat with RFA due to the heat-sink effect. The heat sink effect results from the cooling effect of blood flowing through the tumour, especially in the kidney which is perfused by 20% of the circulating blood volume [9,10]. In addition, this may potentially allow assessment of the treatment effect e.g., quantitative rather than qualitative measurement of the diminished perfusion in the zone of ablation post-RFA. Early accurate diagnosis of residual disease in the ablated area allows prompt re-intervention if clinically indicated. The location of the renal tumour may also influence the perfusion of the tumour and potentially play a role in explaining centrally located tumour often is harder to treat with likelihood of incomplete treatment and exophytic tumour often is more likely to be completely ablated [11].

The aims of this study were to correlate blood flow to the RCC with the RFA treatment time, location of the tumour (exophytic, mixed, parenchymal and central) and tumour size and also to investigate if the early treatment effect of RFA in RCC can be detected with Dynamic Contrast Enhanced-Magnetic Resonance Imaging (DEC-MRI) perfusion measurement.

## 2. Materials and Methods

### 2.1. Patients

Twenty consecutive patients (13 men, 7 women; age range 30–80 years; mean age 69 years) with a total of 21 renal tumours underwent image-guided RFA treatment and were prospectively recruited to the study from May 2010 to November 2011. All had primary RCC and one patient had two renal tumours. Their renal tumours were evaluated with DCE-MRI one week before and at one month after RFA treatment as according to our local institution policy. The imaging review study was granted approval by the local research ethics committee and formal written consent was waived as DCE-MRI is part of the routine clinical imaging for patients undergoing renal ablation in our institution.

### 2.2. Biopsy Procedures and RF Ablation

All RFA procedures were performed under general anaesthesia and as an elective procedure in all patients with a routine admission the day before the procedure. All renal tumour biopsies (two 18 G core needle biopsies with a co-access sheath system (Boston Scientific, Marlborough, MA, USA)) were performed at the time of RF ablation under CT guidance as standard clinical care.

All ablations were performed by a single experienced consultant radiologist (Dr Tze Min Wah). 21 RCCs were ablated with an impedance-controlled pulsed current from a 200-W generator (Boston Scientific, Marlborough, MA, USA). RCC target tissue cell death is achieved via tissue desiccation and loss of the ability to conduct current and subsequent rise in impedance. ‘Roll off’ equates to a clinical endpoint where complete tissue coagulation is reached when the impedance reaches a clinically relevant level and there is concurrent power shut down of the generator. Ablation was performed with a varying size (3, 3.5 or 4 cm) umbrella shaped multi-tines needle electrodes (LeVeen CoAccess RFA needle electrode, Boston Scientific, Marlborough, MA, USA), to match the size of the tumour. During treatment, the number of overlapping ablations was dependent on the size and geometry of the tumour. For this system, the RFA treatment time is the total time required to treat the whole tumour and the timing of individual ablations was impedance-controlled, depending upon the tissue vascularity and resistance. All 21 tumours were ablated under CT guidance in a CT interventional suite as this allows assessment of the safety margin of the surrounding organ in relation to the multi-tines needle electrode. Post-RFA, all patients were admitted for overnight observation and discharged home if clinically stable the following day as per standard care. In this cohort of patients, all patients had a routine 2-day hospital stay.

### 2.3. Dynamic Contrast Enhanced (DCE) MRI

All MRI examinations were performed on a Siemens (Erlangen, Germany) Magnetom Symphony 1.5 T system. The examination was performed at 3.1 days (+/−SD 2.6 days) prior to and at 28.1 days (+/−SD 1.2 days) following RFA treatment of the renal tumours. A dedicated four element body array coil and integrated spine coil were used for signal reception. The MRI examination included T1, T2 and true-fast imaging with steady state precession sequences in the axial, sagittal and coronal planes for morphological imaging.

Perfusion assessment with DCE-MRI was performed in an oblique coronal plane to include both kidneys and a section of the descending aorta [12]. A pre-contrast T1 weighted 3D FLASH breath hold sequence was performed to establish a signal baseline (6 repeats at 2 s intervals). The Magnetic Resonance Imaging sequence parameters were: TR: 2.44 ms, TE: 1.19 ms, flip angle: 18 degrees, FOV: 400 mm × 400 mm × 130 mm, matrix: 192 × 96 × 18 and voxel volume: 63.4 mm^3^.

Dotarem (Gd-DOTA Gadoteric acid, Guerbet, France) at a dose of 0.1 mmol/kg body weight was injected into an antecubital vein at a flow rate of 4 mL/s. Injection of the contrast was chased by 20 mL of saline at 4 mL/s using a MR compatible automated injector. The acquisition of the data was triggered at the same time as the contrast injection. The scanning range and parameters were copied from the pre-contrast breath hold T1 weighted sequence. The DCE-MRI was performed under gentle breathing for a period of 1 min (30 repeats at 2 s intervals). In addition, pre and post contrast T1 weighted volume interpolated breath-hold examination (VIBE) sequences were performed for morphological treatment assessment in both axial and coronal planes.

### 2.4. Data Post Processing

**Figure 1:**
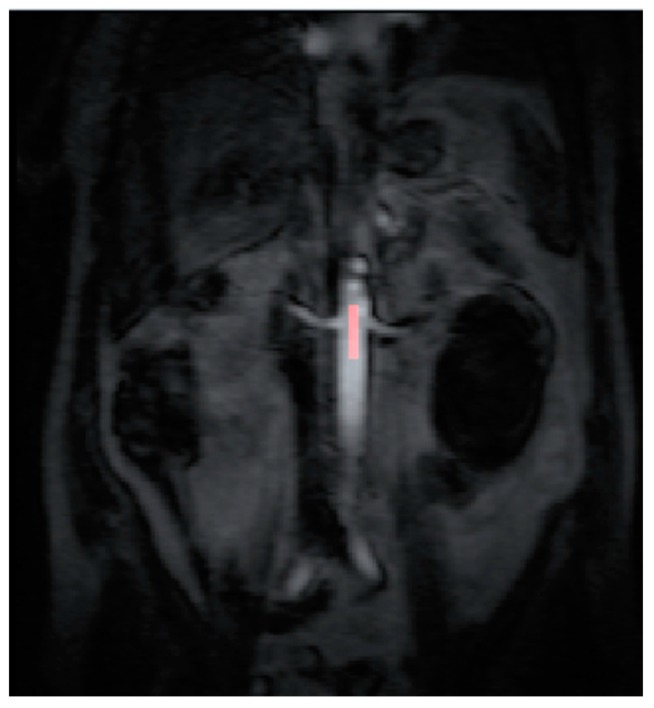
The arterial input function (AIF) region of interest (ROI) was drawn inside the aorta at the approximate level of the origin of the vascular pedicles of the kidneys in the dynamic series.

**Figure 2:**
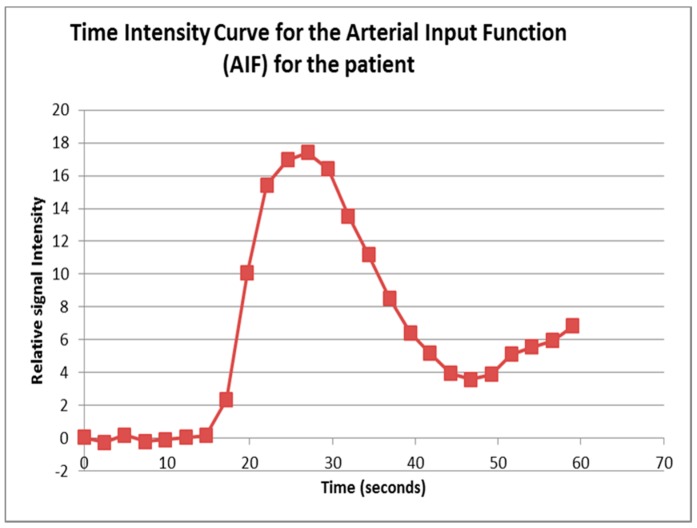
The time intensity curve produced by the Platform for Research in Medical Imaging (PMI) version 0.4 Software for arterial input function (AIF) as outlined by region of interest (ROI) in the aorta as in Figure 1.

All the MR images were anonymized and post processing was performed offline by a single consultant radiologist with greater than 10 years of clinical experience (Dr Tze Min Wah). Images were uploaded into PMI 0.4 (Platform for Research in Medical Imaging Version 0.4 [13], which was running on a desktop PC. A standardized 4-voxel region was used to extract an arterial input function (AIF). This region of interest (ROI) was drawn inside the aorta at the approximate level of the origin of the vascular pedicles of the kidneys (Figure 1 and Figure 2).

**Figure 3:**
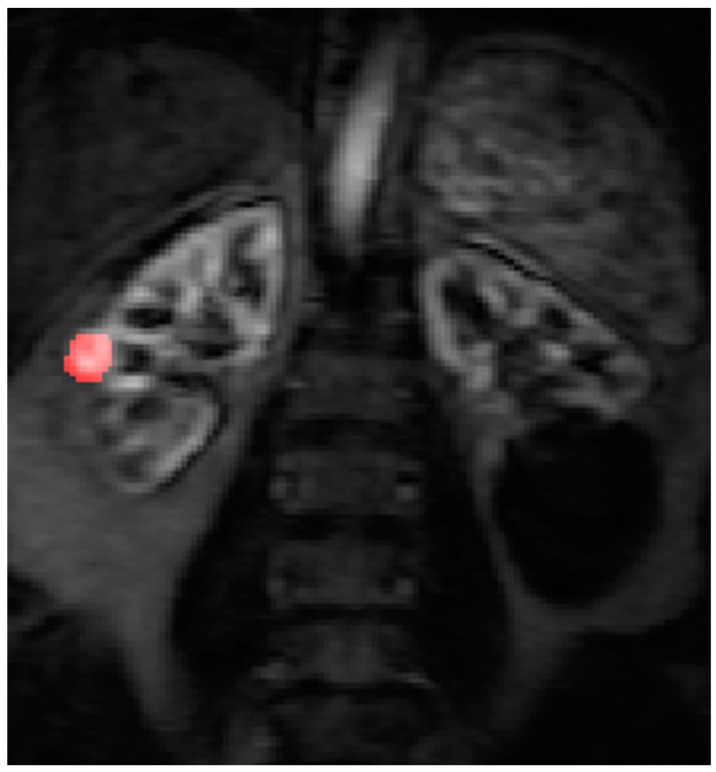
The right renal tumour is sited at the mid polar region of the kidney and there is a large renal cyst at the lower pole of the left kidney. The ROI is drawn on the maximum concentration map generated by Platform for Research in Medical Imaging (PMI) software before RFA treatment.

**Figure 4:**
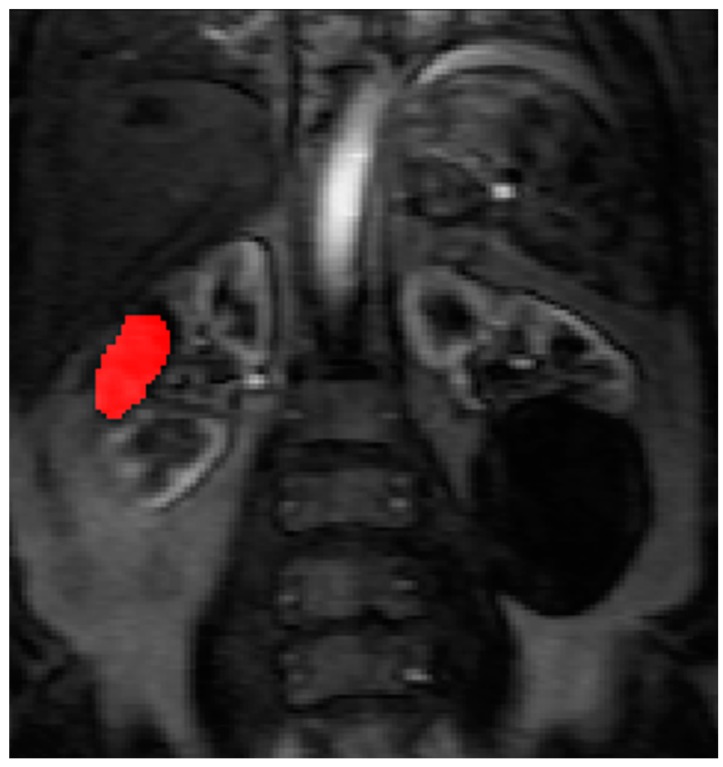
The ROI is drawn on the maximum concentration map generated by PMI software after radiofrequency ablation treatment.

Contrast agent concentration-time curves were approximated using relative change in signal (compared to baseline) against time [13]. To assist in identifying the tumour and drawing the ROI, a map of maximum contrast agent concentration was generated. Using the map, ROIs were drawn to encompass the renal tumour pre RFA (Figure 3) and the whole zone of ablation post RFA (Figure 4).

**Figure 5:**
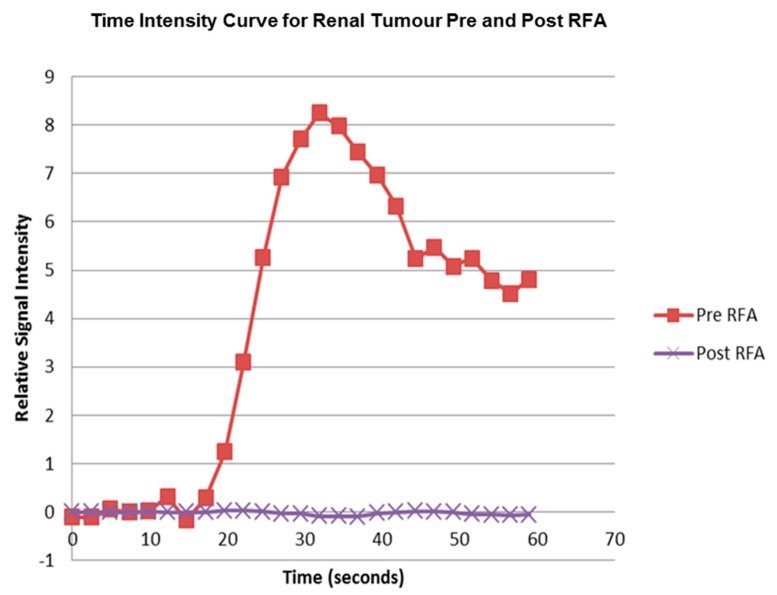
The time intensity curve produced by the PMI 0.4 Software for the right renal tumour as outlined in Figure 2 ROI using the maximum slope technique before and after RFA treatment.

The pre RFA renal tumour and post RFA zone of ablation in every patient were saved and anonymized within the same dataset. Pre RFA, the ROI outlined the bright area which represents the enhancing renal tumour. In this cohort, some of the tumours had a cystic or necrotic component, but only the solid components were included in the ROIs. Post RFA, the ROI outlined the whole zone of ablation which is typically larger than the tumour to allow for the ‘surgical’ margin, this is the dark area on images that represents ‘dead’ renal tumour. If there is any bright area within the zone of ablation, this typically suggests area of enhancement and indicates that there is a viable renal tumour and this would be included with the ROI. This process was repeated for every slice containing pre RFA tumour and the post RFA zone of ablation. The regions for each slice were combined giving a volume of interest that covered the perfused renal tumour in the DCE dataset. This was then analyzed to extract concentration-time curves for the renal tumour before treatment and zone of ablation after treatment (Figure 5). The maximum slope of the tumour curve and the peak value of the AIF were determined.

The initial rise of contrast agent concentration in the tumour, before any contrast agent has reached the veins, is directly related to tumour perfusion. Analysis of the images acquired during the first pass of the contrast agent bolus can be used to estimate tumour perfusion through the use of the maximum slope technique [14].

Tumour perfusion = maximum rate of contrast agent concentration increase in the tumour/peak contrast agent concentration in the AIF (Equation (1))

(1) $$Fb=\frac{\frac{d}{dt}\left[C\left(tumour\right)\right]/max}{\left[C\left(Aorta\right)\right]/max}.$$

*Fb* = Tumour perfusion

*C* (*tumour*) = Time concentration curve for the tumour

*C* (*Aorta*) = Time concentration curve of the aorta

Perfusion of the RCC and the zone of ablation (*Fb*) was calculated using Equation (1) before and after RFA treatment, respectively.

The total blood flow to the tumour was calculated from the volume of the solid enhancing tumour (measured in units of 100 mL tissue) and multiplied by the mean perfusion (mL/min/100 mL tissue).

### 2.5. Testing Intra-Observer Variability

For the assessment of RCC perfusion before treatment, two separate analyses were performed at two independent sittings in order to test for intra-observer variability. A suitable period of time elapsed (>12 weeks) in between the image scoring so that the radiologist could not recall the previous results. The images were presented to the radiologist in a random order and the process of contouring analysis was repeated on the PMI 0.4 Software; it was repeated in a similar reporting condition.

### 2.6. Data Analysis and Statistics

Statistical analysis was performed with Microsoft Excel (2010) software and the software package Stata (Stata Corp. 2009. Stata: release 11. Statistical Software. College Station, TX, USA: Stata Corp. LP). Descriptive statistics (e.g., mean, SD and variance) were reported and differences with a *p* value of less than 0.05 were considered to be statistically significant. The concordance correlation coefficient (r) was used to assess intra-observer variability for the measurement of perfusion for the renal tumour before treatment for the single consultant radiologist. Pre-treatment total blood flow to the renal tumour was correlated with the RFA treatment time using the Spearman rank correlation coefficient. The perfusion of the renal tumour before and after RFA was compared. These paired continuous data were analyzed using a paired *t*-test.

Renal tumour characteristics as volume, perfusion, and tumour location (defined as exophytic, mixed, parenchymal or central) vs. RFA treatment time were analyzed using multivariate analysis to determine their impact on RFA treatment time. The mean RFA treatment time was also correlated with tumour location using the Spearman rank correlation coefficient.

## 3. Results

All the DCE-MRI examinations were completed and evaluated successfully. Twenty one renal tumours were evaluated in 20 patients.

### 3.1. Renal Tumour Morphology and Histology

Seventeen renal tumours were solid and four had cystic components. All were histologically proven RCC. The renal tumour maximum diameter ranged from 1.3 to 4 cm (mean size = 2.5 cm) with tumour volume ranging from 0.5 to 20.7 mL (mean volume = 5.6 mL) and they were all stage T1a renal tumours. Post-RFA, the zone of ablation was larger with volume ranging from 2.5 to 20.8 mL (mean volume = 8.8 mL). In each patient, the total number of overlapping ablations performed ranged from 2 to 4 (mean ablation cycle = 2.6) and the total ablation time was between 7.4 and 63.4 min (mean ablation time = 24.2 min).

### 3.2. Perfusion Measurement: Intra-Observer Variability

Perfusion of renal tumours was measured twice by the single radiologist at two different sittings. The concordance correlation coefficient indicated low intra-observer variability for the measurement obtained (*r* ≥ 0.99, *p* < 0.0001).

### 3.3. Renal Tumour Characteristics vs. RFA Treatment Time

**Figure 6:**
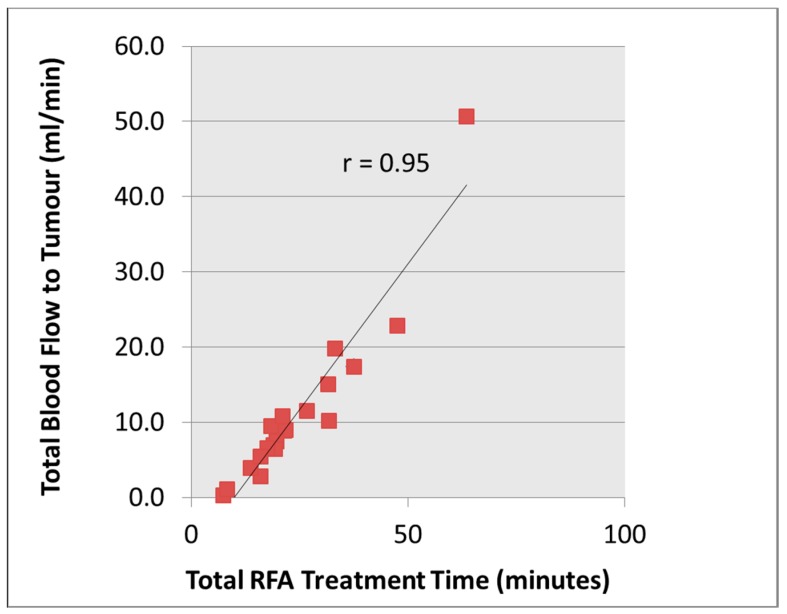
Correlation of total blood flow to the renal tumours and the radiofrequency ablation (RFA) time.

According to the multivariate analysis, the two most important and independent factors that influenced RFA treatment time were the volume and perfusion of the tumour. The tumour volume alone has an r-squared of 87%. When combining both volume and perfusion of the tumour to reflect the tumour vascularity, these made them highly significant (r-squared of 94% and combined correlation coefficient, *r* = 0.97). When these parameters are combined in an estimate of total blood flow there is a very strong correlation with treatment time (*r* = 0.95; Figure 6). In addition, the tumour location was also found to have some relationship with the RFA treatment. The mean RFA treatment time for tumors in different locations was 14.3 min, 22.8 min and 40.9 min for exophytic, mixed and parenchymal locations respectively and the relationship was statistically significant using the Spearman rank correlation coefficient (*ρ* = 0.63, *p* < 0.002).

### 3.4. Perfusion of Renal Tumour: Pre and Post RFA

**Figure 7:**
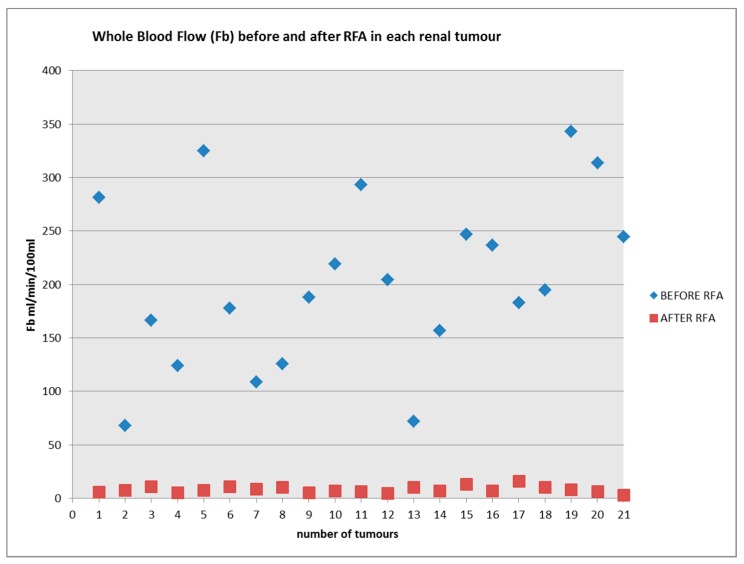
The scatter plot for blood flow in each renal tumour before and after RFA treatment.

Perfusion of the 21 RCCs decreased significantly (*p* < 0.0001) from a mean of 203 (+/−80) mL/min/100 mL pre RFA to 8.1 (+/−3.1) mL/min/100 mL post RFA (Figure 7).

## 4. Discussion

Image-guided RFA of RCC is an established treatment option with good oncological outcome data [15]. In our experience, the treatment time can often be variable and the ability to predict the RFA treatment would help treatment scheduling, since the total treatment time is usually lengthy (3 to 4 h), e.g., the time for induction and recovering from general anesthesia, positioning and intra-procedural preparation of the patient. Other considerations are risk of grounding pad skin burns [16] especially in the elderly with fragile skin when there is prolonged RF treatment time and the potential of inadequate ablative effects. In this pilot study, our hypothesis was that tumours with higher total blood flow would take longer to treat as a result of the heat sink effect. The electrical circuit of the RF generator is controlled by the tissue impedance, and complete desiccation of the tumour tissue will lead to increased tissue resistance and hence circuit cut off by the RF generator system. This study showed that total blood flow to the renal tumour correlated well with the total RFA time (*ρ* = 0.95), which supports our hypothesis.

Using multivariate analysis, the two most important tumour factors that influence the RFA treatment time are perfusion of the tumour and volume of the tumour, which in turn contribute to the total blood flow. In addition, the location of the renal tumour is also found to have a relationship with the mean RFA treatment time; this relationship is statistically significant where the exophytic location has the lowest mean RFA treatment time (14.3 min) whilst the parenchymal location has the highest RFA treatment time (40.9 min) (*ρ* = 0.63, *p* < 0.002). There were no centrally located tumours in this cohort of patients. Other authors have also noted that the renal tumour’s size and location are two important independent predicting factors in determining successful treatment outcome in a single RF ablation [17]. For the exophytic location, it is postulated that it has least heat sink effect from the insulation by the peri-renal fat, which may provide the ‘oven effect’ for effective treatment. However, the relationship was not formally quantified previously and our findings by correlating the location of the renal tumour vs. RFA treatment time have provided the explanation behind this observation.

The study has also demonstrated that perfusion within the zone of ablation decreased significantly (*p* < 0.0001) from a mean of 203 (+/−80) mL/min/100 mL pre RFA to 8.1 (+/−3.1) mL/min/100 mL post RFA in the 21 treated renal tumours. We recognize that though quantitative perfusion measurement is feasible in assessing the treatment effect of RFA in RCC, given the efficacy of the treatment, conventional signal measurement of the MR images may suffice in predicting the majority of the treatment effect. The question remains, however, whether the ability to measure RCC perfusion pre-RFA and assessment of treatment effect post RFA may help to improve the oncologic efficacy/reduce recurrence rate. A larger study with longer term follow up may provide further information into this question.

Unlike solid tumours in some other organs, imaging of renal tumours is challenging as the kidneys move with normal respiration. Breath-holding may be able to counteract this effect but this only allows a very short acquisition approach, which may be sufficient for first pass analysis but is not able to assess extravascular kinetics. In our study, we performed DCE-MRI under free breathing in the oblique coronal plane and there was no significant respiratory motion. All maximum concentration maps generated by the PMI software were suitable for ROI drawing and there was low intra-observer variability in this pilot study for the estimation of renal tumour perfusion before treatment (concordance correlation coefficient; *r* ≥ 0.99, *p* < 0.0001). However, given the small sample size, there remains a potential bias that a single reviewer may still recall details after a long lag time.

There are other models available to estimate RCC perfusion. These include quantitative assessment of various physiological parameters by tracer kinetic modeling e.g., one compartment [18,19] or two compartment analysis [13]. In busy clinical practice, a pragmatic assessment technique is essential and the maximum slope technique with its limited acquisition time and simple analysis was selected for our study [20]. However, there are limitations to the technique. By assuming a linear relationship between relative signal intensity change and contrast agent concentration it is likely that the peak concentration of the AIF will be underestimated [13]. All quantitative methods are affected by this error, but the maximum slope technique is particularly sensitive to it as it only uses the peak value of the AIF. This will result in overestimation of RCC perfusion, but may be somewhat counteracted by venous wash-out of the contrast agent by the time of maximum slope; a well-recognized limitation of the maximum slope technique applied to highly perfused tissues [21]. While the latter weakness is inherent to the maximum slope technique the former could be addressed, with some minor time penalties, by pre-contrast measurement of T1 and a non-linear conversion of signal intensity to contrast agent concentration [18,22].

## 5. Conclusions

It is feasible to measure perfusion of RCC before and the zone of ablation after RFA using DCE-MRI and pre-RFA total tumour blood flow may be used to predict treatment time. Perfusion values are significantly decreased in the zone of ablation, suggesting they may be useful for the assessment of treatment effect. These findings support the argument for a larger study to validate the ability to measure the RCC perfusion pre-RFA and assessment of treatment effect post RFA may help to improve the oncologic efficacy/reduce recurrence rate.

## References

[1] Nguyen M.M., Gill I.S., Ellison L.M. (2006). The evolving presentation of renal carcinoma in the United States: Trends from the Surveillance, Epidemiology, and End Results program. J. Urol..

[2] Russo P., Huang W. (2008). The medical and oncological rationale for partial nephrectomy for the treatment of T1 renal cortical tumors. Urol. Clin. N. Am..

[3] Ljungberg B., Cowan N.C., Hanbury D.C., Hora M., Kuczyk M.A., Merseburger A.S., Patard J.-J., Mulders P.F.A., Sinescu I.C. (2010). EAU guidelines on renal cell carcinoma: The 2010 update. Eur. Urol..

[4] Matin S.F., Ahrar K., Cadeddu J.A., Gervais D.A., McGovern F.J., Zagoria R.A., Uzzo R.G., Haaga J., Resnick M.I., Kaouk J. (2006). Residual and recurrent disease following renal energy ablative therapy: A multi-institutional study. J. Urol..

[5] Wile G.E., Leyendecker J.R., Krehbiel K.A., Dyer R.B., Zagoria R.J. (2007). CT and MR imaging after imaging-guided thermal ablation of renal neoplasms. Radiographics.

[6] Merkle E.M., Nour S.G., Lewin J.S. (2005). MR imaging follow-up after percutaneous radiofrequency ablation of renal cell carcinoma: Findings in 18 patients during first 6 months. Radiology.

[7] Javadi S., Ahrar J.U., Ninan E., Gupta S., Matin S.F., Ahrar K. (2010). Characterization of Contrast Enhancement in the Ablation Zone Immediately after Radiofrequency Ablation of Renal Tumors. J. Vasc. Interv. Radiol..

[8] Karam J.A., Ahrar K., Vikram R., Romero C.A., Jonasch E., Tannir N.M., Rao P., Wood C.G., Matin S.F. (2013). Radiofrequency ablation of renal tumours with clinical, radiographical and pathological results. BJU Int..

[9] Goldberg S.N., Scott Gazelle G., Dawson S.L., Rittman W.J., Mueller P.R., Rosenthal D.I. (1995). Tissue ablation with radiofrequency: Effect of probe size, gauge, duration, and temperature on lesion volume. Acad. Radiol..

[10] Goldberg S.N., Scott Gazelle G., Solbiati L., Rittman W.J., Mueller P.R. (1996). Radiofrequency tissue ablation: Increased lesion diameter with a perfusion electrode. Acad. Radiol..

[11] Gervais D.A., McGovern F.J., Arellano R.S., McDougal W.S., Mueller P.R. (2003). Renal cell carcinoma: Clinical experience and technical success with radio-frequency ablation of 42 tumors. Radiology.

[12] Buckley D.L., Shurrab A.E., Cheung C.M., Jones A.P., Mamtora H., Kalra P.A. (2006). Measurement of single kidney function using dynamic contrast-enhanced MRI: Comparison of two models in human subjects. J. Magn. Reson. Imaging.

[13] Notohamiprodjo M., Sourbron S., Staehler M., Michaely H.J., Attenberger U.I., Schmidt G.P., Boehm H., Horng A., Glaser C., Stief C. (2010). Measuring perfusion and permeability in renal cell carcinoma with dynamic contrast-enhanced MRI: A pilot study. J. Magn. Reson. Imaging.

[14] Miles K.A. (2003). Functional CT imaging in oncology. Eur. Radiol..

[15] Psutka S.P., Feldman A.S., McDougal W.S., McGovern F.J., Mueller P., Gervais D.A. (2013). Long-term oncologic outcomes after radiofrequency ablation for T1 renal cell carcinoma. Eur. Urol..

[16] Rhim H., Dodd G.D., Chintapalli K.N., Wood B.J., Dupuy D.E., Hvizda J.L., Sewell P.E., Goldberg S.N. (2004). Radiofrequency thermal ablation of abdominal tumors: Lessons learned from complications. Radiographics.

[17] Gervais D.A., Arellano R.S., McGovern F.J., McDougal W.S., Mueller P.R. (2005). Radiofrequency ablation of renal cell carcinoma: Part 2, Lessons learned with ablation of 100 tumors. Am. J. Roentgenol..

[18] Tofts P.S., Kermode A.G. (1991). Measurement of the blood-brain barrier permeability and leakage space using dynamic MR imaging. 1. Fundamental concepts. Magn. Reson. Med..

[19] Brix G., Semmler W., Port R., Schad L.R., Layer G., Lorenz W.J. (1991). Pharmacokinetic parameters in CNS Gd-DTPA enhanced MR imaging. J. Comput. Assist. Tomogr..

[20] Chapman S.J., Wah T.M., Sourbron S.P., Buckley D.L. (2013). The effects of cryoablation on renal cell carcinoma perfusion and glomerular filtration rate measured using dynamic contrast-enhanced MRI: A feasibility study. Clin. Radiol..

[21] Brix G., Zwick S., Griebel J., Fink C., Kiessling F. (2010). Estimation of tissue perfusion by dynamic contrast-enhanced imaging: Simulation-based evaluation of the steepest slope method. Eur. Radiol..

[22] Hahn O.M., Yang C., Medved M., Karczmar G., Kistner E., Karrison T., Manchen E., Mitchell M., Ratain M.J., Stadler W.M. (2008). Dynamic contrast-enhanced magnetic resonance imaging pharmacodynamic biomarker study of sorafenib in metastatic renal carcinoma. J. Clin. Oncol..

